# Detrending the Waveforms of Steady-State Vowels

**DOI:** 10.3390/e22030331

**Published:** 2020-03-13

**Authors:** Marnix Van Soom, Bart de Boer

**Affiliations:** Artificial Intelligence Laboratory, Vrije Universiteit Brussel, Pleinlaan 2, 1050 Brussels, Belgium; bart.de.boer@ai.vub.ac.be

**Keywords:** formant, steady-state, vowel, detrending, acoustic phonetics, source-filter theory, probability theory, uncertainty quantification, model averaging, nested sampling

## Abstract

Steady-state vowels are vowels that are uttered with a momentarily fixed vocal tract configuration and with steady vibration of the vocal folds. In this steady-state, the vowel waveform appears as a quasi-periodic string of elementary units called pitch periods. Humans perceive this quasi-periodic regularity as a definite pitch. Likewise, so-called pitch-synchronous methods exploit this regularity by using the duration of the pitch periods as a natural time scale for their analysis. In this work, we present a simple pitch-synchronous method using a Bayesian approach for estimating formants that slightly generalizes the basic approach of modeling the pitch periods as a superposition of decaying sinusoids, one for each vowel formant, by explicitly taking into account the additional low-frequency content in the waveform which arises not from formants but rather from the glottal pulse. We model this low-frequency content in the time domain as a polynomial trend function that is added to the decaying sinusoids. The problem then reduces to a rather familiar one in macroeconomics: estimate the cycles (our decaying sinusoids) independently from the trend (our polynomial trend function); in other words, detrend the waveform of steady-state waveforms. We show how to do this efficiently.

## Relation of This Work to the Conference Paper

We have already presented the main idea of this work in a preceding conference paper [1], albeit in a relatively obscure form. In this work we give an improved theory together with a more complete picture, by showing how that main idea, the detrending of steady-state vowel waveforms, can be derived heuristically from canonical source-filter theory in a simple way.

As this work examines only steady-state systems, we need only a quite limited set of concepts from acoustic phonetics to get by—which, in addition, are well defined by virtue of the assumed steady-state. To make this work self-contained, we introduce these concepts where needed, though more tersely compared to the conference paper. We refer the reader to the conference paper and the references given for more details.

## 1. Introduction

Formants are characteristic frequency components in human speech that are caused by resonances in the vocal tract (VT) during speech production and occur both in vowels and consonants (In the literature, the distinction between the physical resonance of the VT and the associated characteristic frequency in the resulting speech is often not made [2] (p. 179); as such, the term “formant” can mean both, and we will follow that custom here). In source-filter theory [3], the “standard model” of acoustic phonetics, the speech production process is modeled as a linear time-invariant system [4]. In a nutshell, the input to the system is the glottal source (i.e., the vibration of the vocal folds), the system’s transfer function describes the formants of the VT by assigning one conjugate pole pair to each formant, and the output is the speech signal. The speech signal is thus the result of filtering the glottal source signal with the VT formants.

The formants’ bandwidth, frequency and relative intensity can be manipulated by us humans through changing the VT configuration (such as rounding the lips or closing the mouth) during speech. Measuring the formants of a given speech fragment is a routine preoccupation in the field of acoustic phonetics as formants can be said to carry basic information—above all to human listeners—about uttered phonemes [5], speaker sex, identity and physique [6], medical conditions [7], etc.

Accordingly, when formants are used as one of the pieces of information by a speech processing computer program trying to determine, say, the height of a speaker [8], it is desirable to acknowledge the uncertainty in the formant measurement, as this uncertainty propagates to the uncertainty about the speaker’s height. While our contention that such uncertainty quantification is desirable stems mainly from a principled point of view [9], we argue that in critical cases such as forensic speaker identification [10], the ability to assign a degree of confidence to formant measurements—upon which further conclusions rest—is valuable, perhaps essential, and well worth the considerable extra computational effort required (As far as we know, while “there is a huge and increasing demand for [forensic speaker identification] expertise in courts” [10] (p. 255), uncertainty quantification for formant measurements is currently not in (widespread) use in forensics [10,11,12]. We are aware of several works on quantifying and discussing the nature of the variability and reliability of formant measurements that have been published quite recently [2,13,14,15,16,17]; this matter is discussed further in the conference paper under the umbrella term “the formant measuring problem”). In more routine circumstances one may simply take the error bars on the formant estimates as a practical measure of the computer program’s trust in its own output. As with many things, the use for error bars or confidence intervals for formant measurements depends strongly on the application and available resources at hand.

The goal of this paper is a discussion of a simple pitch-synchronous linear model of steady-state vowels capable of quantifying the uncertainty of the formant measurements in a very straightforward way: by *inferring* them in the context of (Bayesian) probability theory [18]. The model, which works by effectively detrending the waveforms of steady-state vowels, is a generalization of previous work by others [19,20] and is similar in principle to [21]’s Bayesian method to infer the fundamental frequency. While the remainder of this Introduction sketches the background and rationale for the model in some detail, readers may wish to skip directly to Section 2 for its actual mathematical statement.

### 1.1. Background

**Figure 1:**
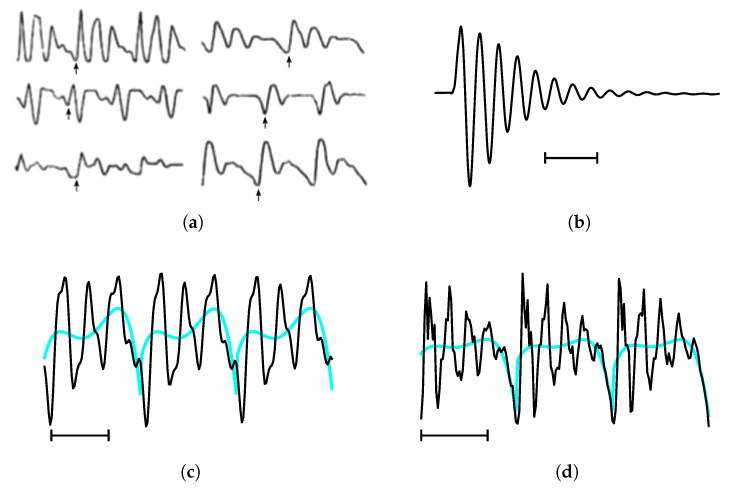
Several illustrations of pitch periods (a,c,d) and a related concept, the impulse response (b). The horizontal lines below the waveforms in (b–d) indicate a duration of 5 ms. The inferred trends in (c,d) are plotted in cyan. (**a**) In 1889, Hermann [26] used Fourier transforms of single pitch periods of steady-state vowels to calculate their spectra, and coined the term “formant” to designate the peak frequencies which were characteristic to the vowel [27] (p. ix). Shown here are examples of steady-state vowels from Hermann’s work. Small vertical arrows indicate the start (glottal closing instant or GCI) of the second pitch period. Adapted from [27] (p. ix); originally from [28] (p. 40). (**b**) The impulse-like response produced when one of the authors excited his vocal tract by flicking his thumb against his larynx whilst mouthing “o”. This is an old trick to emulate the impulse response of the vocal tract normally brought about by sharp GCIs (also known as glottal closures). The impulse responses triggered by sharp GCIs can be observed occasionally in the waveforms of vocal fry sections or as glottal stops /ʔ/ [27] (p. 49). (**c**) Three pitch periods taken from a synthesized steady-state instance of the vowel /ɤ/ at a fundamental frequency of 120 Hz and sampled at 8000 Hz. The trend inferred by our model is a fifth order polynomial. This example is discussed in Section 4.1. (**d**) Three pitch periods taken from a steady-state instance of the vowel /æ/ at a fundamental frequency of 138 Hz. The trend inferred by our model is a weighted combination of a 4th and 5th order polynomial. This example is discussed in Section 4.2. Source: [29], bdl/arctic_a0017.wav, 0.51–0.54 s, resampled to 8000 Hz.

Historically, formants and steady-state vowels (as opposed to vowels in general) are intrinsically connected because the concept of a formant was originally defined in terms of steady-state vowels—see Figure 1a. What we mean by steady-state vowels in this work is the steady-state portion (which may never be attained in some cases) of a vowel utterance. This is the time interval in which (a) the VT configuration can be taken to be approximately fixed, leading to essentially constant formants, and (b) the vocal folds are sustained in a reasonably steady vibration called the glottal cycle, during which the vocal folds open and close periodically. Because of (a) and (b) the vowel waveform appears as a quasi-periodic string of elementary units called pitch periods (We use “quasi-periodic” in the (colloquial) sense of Reference [22] (p. 75), i.e., designating a recurrent function of time for which the waveforms for successive periods are approximately the same. Examples are given in Figure 1a,c,d). In practice, steady-state vowels are identified simply by looking for such quasi-periodic strings, which typically consist of about 3 to 5 pitch periods [5]. Results from clinical trials [23] indicate that normal (non-pathological) voices are usually Type I [24], i.e., phonation results in nearly periodic sounds, which supports the notion that uttered vowels in normal speech typically reach a steady-state before “moving on” [25].

During the steady-state, the pitch periods happen in sync with the glottal cycle [30]. The start of the pitch period coincides with the glottal *closing* instant (GCI), which causes a sudden agitation in the waveform and can be automatically detected quite reliably [31]. The duration between the GCIs, i.e., the length of the pitch periods, defines the fundamental frequency of the vowel, which for women is on the order of ${\left(5\;\mathrm{ms}\right)}^{-1}=200\;\mathrm{Hz}$ and for men on the order of ${\left(8\;\mathrm{ms}\right)}^{-1}=125\;\mathrm{Hz}$ [32,33]. The GCI pulse at the start of each pitch period is often so sharp that, recalling source-filter theory, the resulting response of the VT approximates the VT impulse response—see Figure 1b. In contrast, the glottal *opening* instant (GOI) excites the VT only weakly, which injects additional low-frequency content into the waveform roughly halfway through the pitch period.

### 1.2. The Pinson Model

The observation that the GCI pulse is often sufficiently sharp underlies Pinson’s basic model [19] of the portion of the pitch period *between GCI and GOI* as being essentially the VT impulse response. The model was originally proposed to estimate formants during voiced speech directly in the time domain. The reason why the model does not address the whole pitch period is an additional complication due to the GOI: when the glottis is open, the VT is coupled to the subglottal cavities (such as the lungs), which slightly increases the bandwidths of the formants, and thus slightly shifts the poles of the VT transfer function. If the “closed half” of the pitch period is characterized by *Q* formants with bandwidths $\alpha =\{\alpha_{1}\cdots \alpha_{Q}\}$ and frequencies $\omega =\{\omega_{1}\cdots \omega_{Q}\}$, then the VT transfer function $H_{P}$ has *Q* conjugate pole pairs and up to (2Q−1) zeros:

(1) $$H_{P}\left(s,\alpha ,\omega \right)=\frac{N(s)}{\prod_{k=1}^{Q}\left[s-\left(\alpha_{k}+i\omega_{k}\right)\right]\left[s-\left(\alpha_{k}-i\omega_{k}\right)\right]}\;\left(\deg\left(N\left(s\right)\right)<2Q\right),$$

where N(s) is a polynomial of constrained degree in order that $H_{P}$ be proper. (Note that, to emphasize the physical connection to resonances, we denote the bandwidth and frequency of the *k*th formant by $\left(\alpha_{k},\omega_{k}\right)$ and not by $\left(B_{k},F_{k}\right)$ as is more customary in acoustic phonetics.)

Pinson’s model for the “closed half” of one pitch period is then just a bias term plus the associated impulse response of $H_{P}$ (its inverse Laplace transform) constrained to live between GCI (t=0) and GOI ($t=T_{O}$):

(2) $$f_{P}\left(t,b,\alpha ,\omega \right)=b_{1}+\sum_{k=1}^{Q}\left(b_{k+1}\cos\omega_{k}t+b_{k+1+Q}\sin\omega_{k}t\right)\exp\{-\alpha_{k}t\}\;\left(0\leq t<T_{O}\right).$$

Here the (2Q+1) amplitudes $b=\{b_{1}\cdots b_{1+2Q}\}$ are determined by N(s), α and ω through the partial fraction decomposition of Equation (1).

The model is then put to use by locating a steady-state vowel, choosing the central pitch period and sampling the “closed half” between GCI and GOI (which at the time had to be estimated by hand). Denoting the *N* samples by $d=\{d_{1}\cdots d_{N}\}$ sampled at times $t=\{t_{1}\cdots t_{N}\}$, estimates of the *Q* formants were found by weighted least-squares:

$$\left(\hat{b},\hat{\alpha },\hat{\omega }\right)={\mathrm{argmin}}_{\left(b,\alpha ,\omega \right)}\sum_{i=1}^{N}w_{i}^{2}{\left[d_{i}-f_{P}\left(t_{i},b,\alpha ,\omega \right)\right]}^{2}.$$

Here $w_{i}$ is an error-weighting function which deemphasizes samples close to GCI and GOI. The amplitude estimates $\hat{b}$ can be used to reconstruct the fit to the data and determine N(s) but are otherwise not used for the formant estimation.

Two features of Pinson’s model are of interest here. The first one is that the bandwidth estimates obtained by this method seem to be (much) more reliable than those obtained by today’s standard linear predictive coding (LPC) methods [13,14], when compared to bandwidths measured by independent methods [4,34,35]. The second one is the direct parametrization of the model function in Equation (2) by the formant bandwidths and frequencies (α,ω), which, as we explain in Section 3, transparently enables uncertainty quantification for their estimates $\left(\hat{\alpha },\hat{\omega }\right)$ in a straightforward and transparent way—this is much harder in LPC-like methods.

### 1.3. The Proposed Model for a Single Pitch Period

The model for a single pitch period we propose in this paper is a simple generalization of Pinson’s model in Equation (2) based on the empirical observation that the waveforms in pitch periods often seem to oscillate around a baseline or *trend*, which becomes more pronounced towards the end of the pitch period—see the examples in Figure 1c–d. In order to model this trend, we generalize the bias term in Equation (2) to an arbitrary polynomial of order P−1 and widen the scope of the model to the *full* pitch period of length *T*:

(3) $$f\left(t,b,\alpha ,\omega \right)=\sum_{k=1}^{P}b_{k}t^{k-1}+\sum_{k=1}^{Q}\left(b_{k+P}\cos\omega_{k}t+b_{k+P+Q}\sin\omega_{k}t\right)\exp\{-\alpha_{k}t\}\;\left(0\leq t<T\right).$$

As before, the $b=\{b_{1}\cdots b_{P+2Q}\}$ are the model amplitudes, but now *f* is an implicit function of (P,Q), the *model orders*. *P* and *Q* will be subject to variation during model fitting, as opposed to the Pinson model where P≡1 and *Q* was decided upon beforehand. The Bayesian approach in Section 3 avoids such a particular choice by using *model averaging* over all allowed values of (P,Q) to estimate the 2Q formant bandwidths and frequencies (α,ω).

As we will discuss in Section 2.4, the trend is caused by the weak excitation of the VT by the GOI. The trend is essentially a low-frequency byproduct of the glottal “open-close” cycle driving steady-state vowels (and is therefore absent at isolated glottal closure events, as in Figure 1b). The main innovation of the model is the assumption that the low-frequency content can be modeled adequately by superimposing a polynomial to the impulse response of the VT in the time domain. This reduces the problem of estimating formants to one frequently encountered in macroeconomics. This problem is the detrending of nonstationary time series such as business cycles for which one needs to estimate the cycles (in our case, parametrized by α and ω) independently from the trend (in our case $\sum_{k=1}^{P}b_{k}t^{k-1}$) [36].

Since our proposed model for a single pitch period in Equation (3) is a generalization of Equation (2), it inherits the more reliable bandwidth estimation and the ability for straightforward uncertainty quantification from the Pinson model. In addition, the cumbersome labor of handpicking the “closed half” of the pitch period is eliminated by extending the scope of the model to full pitch periods, for which automatically estimated GCIs can be used. However, we pay for this convenience with a less precise model as we do not take into account the change in the formant bandwidths during the open part of the glottal cycle.

### 1.4. Outline

In Section 2 we state the model for a steady-state vowel, which is a chain of single pitch period models all sharing the same (α,ω) parameters, and discuss the origin of the trend.

In Section 3 we discuss how the formants and the uncertainty on their estimates are inferred using Bayesian model averaging and the nested sampling algorithm [37].

In Section 4 we apply the model to synthetic data and to real data.

Section 5 concludes.

## 2. A Pitch-Synchronous Linear Model for Steady-State Vowels

The model we propose is a simple variation of the standard linear model [38]:

(4) $$d=f\left(t,b,\theta \right)+e=G\left(t,\theta \right)\;b+e\;\mathrm{where}\;e\;∼N\left(0,\sigma^{2}I\right).$$

Here $d=\{d_{1}\cdots d_{N}\}$ is a vector holding the dataset of *N* points sampled at times $t=\{t_{1}\cdots t_{N}\}$, f is the model function, G is an N×m matrix holding the *m* basis functions which are function of t and the *r* “nonlinear” parameters θ, and $b=\{b_{1}\cdots b_{m}\}$ is a vector of *m* “linear” amplitudes. (The variables just listed describe the standard linear model in general terms; we connect these variables to our specific problem below in Section 2.1) The probabilistic aspect enters with our pdf for the vector of *N* errors $e=\{e_{1}\cdots e_{N}\}$ which is the classical separable multivariate Gaussian characterized by a single parameter, the noise amplitude σ (For more on the rationale behind assigning this pdf, see References [39,40] or, more concisely, Reference [41]). The noise power $\sigma^{2}$ may be also be expressed as the signal-to-noise ratio $\mathrm{SNR}=10\log_{10}f^{T}f/N\sigma^{2}$.

It is well-known that for certain priors the simple form in Equation (4) allows for the marginalization over the amplitudes b and noise amplitude σ in the posterior distribution p(b,θ,σ|d,I) (where I≡ourpriorinformation), such that the posterior for the *r* nonlinear parameters p(θ|d,I) can be written in closed form.

The variation on the standard linear model just mentioned consists of promoting the dataset to a set of *n* dataset vectors, one for each pitch period in the steady-state vowel,

$$d\to \left\{d_{1}\cdots d_{n}\right\},\;t\to \left\{t_{1}\cdots t_{n}\right\},$$

and we fit the model to each $d_{i}$ simultaneously while keeping θ and σ fixed but allowing each pitch period its own set of *m* amplitudes $b_{i}$ and errors $e_{i}$. Thus Equation (4) becomes the set of equations:

(5) $$d_{i}=f\left(t_{i},b_{i},\theta \right)+e_{i}=G\left(t_{i},\theta \right)\;b_{i}+e_{i}\;\mathrm{where}\;\{\begin{array}{cc} i=1\cdots n & \mathrm{is}\;\mathrm{the}\;\mathrm{pitch}\;\mathrm{period}\;\mathrm{index}\\ e_{i}∼N\left(0,\sigma^{2}I\right) & \end{array}$$

The form of Equation (5) and our choice of priors below ensure that the marginalization over the $\left\{b_{i}\right\}$ and σ is still possible. What remains is to specify the model function f and the priors for the $\left\{b_{i}\right\}$, θ and σ [42].

### 2.1. The Model Function

Given a steady-state vowel $\left\{d_{1}\cdots d_{n}\right\}$ segmented into *n* pitch periods, typically with the help of an automatic GCI detector. We assume that the data have been normalized and sampled at regular intervals and choose dimensionless units, such that the sampling times are $t_{i}=\left\{0,1,\ldots ,N_{i}-1\right\}$ where $N_{i}$ is the length of the *i*th pitch period.

If we assume that the steady-state vowel is characterized by *Q* formants, the r=2Q nonlinear parameters of the model are the *Q* formant bandwidths $\alpha =\{\alpha_{1}\cdots \alpha_{Q}\}$ and *Q* formant frequencies $\omega =\{\omega_{1}\cdots \omega_{Q}\}$, i.e.,

$$\theta =\left(\alpha ,\omega \right)\equiv \left(\alpha_{1},\ldots ,\alpha_{Q},\omega_{1},\ldots ,\omega_{Q}\right).$$

Further assuming that the trend in each pitch period can be modeled by a polynomial of degree P−1 (which may differ in shape in each pitch period but always has that same degree), the model function for the *i*th pitch period has m=P+2Q basis functions and the same amount of amplitudes $b_{i}$:

(6) $$f\left(t_{i},b_{i},\theta \right)=G\left(t_{i},\theta \right)\;b_{i}\equiv G_{i}\;b_{i},$$

where $G_{i}$ is an $N_{i}\times m$ matrix holding the *P* polynomials and 2Q damped sinusoids:

(7) $${\left[G_{i}\right]}_{jk}=\{\begin{array}{cc} j^{k-1} & \left(1\leq k\leq P\right)\\ \cos\left(j\omega_{l}\right)\exp-j\alpha_{l} & \left(P<k\leq P+Q\;\mathrm{while}\;l=1\cdots Q\right)\\ \sin\left(j\omega_{l}\right)\exp-j\alpha_{l} & \left(P+Q<k\leq P+2Q\;\mathrm{while}\;l=1\cdots Q\right) \end{array}$$

It is easy to verify that Equations (6) and (7) together are equivalent to the model for a single pitch period in Equation (3) we have motivated in Section 1.3. Thus, to summarize, our pitch-synchronous linear model for a steady-state vowel is essentially a chain of *n* linear single pitch period models, all constrained by (or rather, frustrated into) sharing the same parameters θ, which embodies our steady-state assumption that the formants do not change appreciably. The trend functions are only constrained by their degree, such that their shape may vary from period to period. In addition, the amplitudes of the damped sines may vary as well, which means the intensity and phase of the damped sines can vary from period to period.

### 2.2. The Priors

The prior pdfs for the model parameters are

$$\begin{array}{ccc} p(\left\{b_{1}\cdots b_{n}\right\}|I) & =\prod_{i=1}^{n}{\left(2\pi \delta^{2}\right)}^{-m/2}\exp\{-\frac{{b_{i}}^{T}b_{i}}{2\delta^{2}}\} & \\ & ={\left(2\pi \delta^{2}\right)}^{-nm/2}\exp\{-\frac{\sum_{i=1}^{n}{b_{i}}^{T}b_{i}}{2\delta^{2}}\}\; & \left(b_{i}\in R^{m}\right) \end{array}$$

$$\begin{array}{ccc} p(\theta |I) & =\prod_{k=1}^{r}{\left[\log\theta_{k}^{\mathrm{hi}}/\theta_{k}^{\mathrm{lo}}\right]}^{-1}\frac{1}{\theta_{k}}\; & \left(\theta_{j}^{\mathrm{lo}}<\theta_{j}<\theta_{j}^{\mathrm{hi}}\right) \end{array}$$

$$\begin{array}{ccc} p(\sigma |I) & ={\left[\log\sigma^{\mathrm{hi}}/\sigma^{\mathrm{lo}}\right]}^{-1}\frac{1}{\sigma }\; & \left(\sigma^{\mathrm{lo}}<\sigma <\sigma^{\mathrm{hi}}\right) \end{array}$$

The pdfs are zero outside of the ranges indicated between the parentheses on the right.

In our implementation of the model we set δ=1 because (a) we normalize the data before fitting the model and (b) we use normalized Legendre functions instead of the computationally inconvenient polynomial functions in Equation (7) [43]. Thus this assignment for $p(\left\{b_{i}\right\}|I)$ essentially expresses that we expect all amplitudes to be of order one and acts like a regularizer such that the amplitudes take on “reasonable” values [44].

The Jeffreys priors for the formant bandwidths and frequencies θ stem from representation invariance arguments reflecting our a priori ignorance about their true values—see in App. A in [39]. The ranges $\left[\theta_{j}^{\mathrm{lo}},\theta_{j}^{\mathrm{hi}}\right]$ in which the true values are supposed to lie must be decided by the user. Typically, the user can estimate the θ directly with LPC. Additionally, based on the phoneme at hand, one can look up plausible ranges for the formant frequencies and bandwidths in the literature, e.g., References [4,45]. In our experiments in Section 4 we used both approaches to set the ranges for θ (see Table 1 below) [46].

Likewise, the Jeffreys prior for the noise amplitude σ is bounded by $\sigma^{\mathrm{lo}}$ and $\sigma^{\mathrm{hi}}$. For the lower bound we may take $\sigma^{\mathrm{lo}}\ll 1$ (e.g., on the order of the quantization noise amplitude) and for the upper bound we would conceivably have $\sigma^{\mathrm{hi}}\approx 1$ because of the normalization imposed on the data. As we will discuss below, the precise values of these bounds have a negligible influence on our final conclusions (i.e., model averaging over values of (P,Q) and obtaining weighted samples from the posterior of θ) on the condition that they may be taken sufficiently wide such that most of the mass of the integral below in Equation (20) is contained within them.

### 2.3. The Likelihood Function

The likelihood function is

(8) $$\begin{array}{cc} L(\left\{b_{1}\cdots b_{n}\right\},\theta ,\sigma ) & =p(\left\{d_{1}\cdots d_{n}\right\}|\left\{b_{1}\cdots b_{n}\right\},\theta ,\sigma ,I)\\ \; & ={\left(2\pi \sigma^{2}\right)}^{-N/2}\exp\{-\frac{Q_{F}}{2\sigma^{2}}\}, \end{array}$$

where $N=\sum_{i=1}^{n}N_{i}$ and the scalar “least-squares” quadratic form

$$Q_{F}=\sum_{i=1}^{n}{e_{i}}^{T}e_{i}=\sum_{i=1}^{n}{\left(d_{i}-G_{i}\;b_{i}\right)}^{T}\left(d_{i}-G_{i}\;b_{i}\right).$$

### 2.4. The Origin of the Trend

In this Section we present a heuristic derivation of the trend from source-filter theory.

According to the source-filter model of speech, the speech wave y(t) is in general the output of a linear time-invariant system which models the VT with impulse response (transfer function) h(t) (H(s)) and the radiation characteristic with impulse response (transfer function) r(t) (R(s)), and is driven by the glottal flow u(t):

y(t)=u(t)∗h(t)∗r(t),

where * denotes convolution.

We now proceed in the canonical way. As R(s)∝s for up to 4 kHz [4] (p. 128), and we work modulo rescaling, we can take [47]

(9) $$y\left(t\right)≃u^{\prime }\left(t\right)*h\left(t\right)$$

in this frequency range; accordingly, the glottal source may be taken to be the derivative of the glottal flow $u^{\prime }\left(t\right)$. In this paper we will always work with signals resampled to a bandwidth of 4 kHz, which essentially limits us to the first three or four formants [48] (p. 20).

If we decompose the glottal cycle into a sharp delta-like excitation at GCI and a weak excitation at GOI, the glottal source during a pitch period may be written as

(10) $$u^{\prime }\left(t\right)\approx a_{1}\delta \left(t\right)+l\left(t\right),$$

where $a_{1}$ is a constant, and l(t) represents a slowly changing and broad function in which the spectral power |L(ω)| decreases quickly with increasing frequency ω. Substituting Equation (10) in Equation (9) gives

$$y\left(t\right)≃u^{\prime }\left(t\right)*h\left(t\right)\approx \left(a_{1}\delta \left(t\right)+l\left(t\right)\right)*h\left(t\right)=a_{1}h\left(t\right)+l\left(t\right)*h\left(t\right),$$

where, as before, y(t) represents the speech signal during one pitch period.

As l(t) is a smooth and broad function, the magnitude of its Fourier transform |L(ω)| will be quite narrowly concentrated around ω≈0. In contrast, |H(iω)| will have its first peak only at $F_{1}\gg 0$, the first formant, and therefore generally only a slowly rising slope near frequencies ω≈0 if H(s) can be represented as an all-pole transfer function (which is the assumption behind LPC [49]). Since

$$l\left(t\right)*h\left(t\right)=L^{-1}\left[L\left(\omega \right)\;H\left(i\omega \right)\right]\left(t\right),$$

where L denotes the Fourier transform, if |L(ω)| falls off sufficiently fast, the slope of |H(iω)| near ω≈0 will be near constant, and we may write

$$L\left(\omega \right)\;H\left(i\omega \right)\approx a_{2}L\left(\omega \right),$$

where $a_{2}=H\left(0\right)$ is a real constant.

Thus we may write, very roughly, that the speech signal during one pitch period

$$y\left(t\right)≃u^{\prime }\left(t\right)*h\left(t\right)\approx \left(a_{1}\delta \left(t\right)+l\left(t\right)\right)*h\left(t\right)=a_{1}h\left(t\right)+a_{2}l\left(t\right).$$

As we have assumed that l(t) is a smooth and broad function, it is reasonable to assume that it can be modeled as a polynomial. Thus l(t) is our trend function, which modulo scaling essentially passes unscathed through convolution with h(t) because of the absolute mismatch in their frequency content.

## 3. Inferring the Formant Bandwidths and Frequencies: Theory

The posterior distribution for the (nm+r+1) model parameters is

(11) $$p\left(\left\{b_{i}\right\},\theta ,\sigma |\left\{d_{i}\right\},I\right)=L\left(\left\{b_{i}\right\},\theta ,\sigma \right)\;p\left(\left\{b_{i}\right\}|I\right)\;p\left(\theta |I\right)\;p\left(\sigma |I\right).$$

We use the nested sampling algorithm [37] to infer the parameters of interest, the formant bandwidths and frequencies θ in the following way:

Once the data $\left\{d_{i}\right\}$ are gathered, a “grid” of plausible model order values and prior ranges for the θ are proposed. Each point (P,Q) on that grid parametrizes a particular model for the $\left\{d_{i}\right\}$. The *evidence for a (P,Q) model*

(12) $$\begin{array}{cc} Z(P,Q) & =p(\left\{d_{i}\right\}|P,Q,I)\\ \; & =\int db_{1}\cdots db_{n}\int d\theta \int d\sigma \;L\left(\left\{b_{i}\right\},\theta ,\sigma \right)\;p\left(\left\{b_{i}\right\}|I\right)\;p\left(\theta |I\right)\;p\left(\sigma |I\right), \end{array}$$

where the integrand is a function of the model orders implicitly, can be estimated with the nested sampling algorithm. A (highly desirable) byproduct of this estimation is the acquisition of a set of weighted samples from the posterior in Equation (11), from which the estimates and error bars of the formant bandwidths and frequencies can be calculated, as well as any other function of them (such as the VT transfer function). However, we show in Section 3.1 that by performing the integrals over $\left\{b_{i}\right\}$ and σ Equation (12) can be written as

(13) $$Z\left(P,Q\right)=\int d\theta \;L_{I}\left(P,Q,\theta \right)\;p\left(\theta |I\right),$$

where $L_{I}$ is the *integrated likelihood*. Thus we can sample our parameters of interest, the θ, directly from Equation (13) instead of Equation (12).

The uncertainty quantification for the formants is then accomplished through Bayesian model averaging. Using Equation (13), the evidence Z(P,Q) is calculated and samples $\theta_{P,Q}^{\left(l\right)}$ with weights $w_{P,Q}^{\left(l\right)}$ are gathered for all allowed (P,Q) values (all values on the grid). Then the formant bandwidth and frequency estimates are calculated from the first (M=1) and second (M=2) moments of the samples through model averaging over (P,Q) (though in practice only one to two values of (P,Q) dominate). Assuming uniform priors for the model orders (i.e., p(P,Q|I)∝1),

(14) $$p\left(\theta |\left\{d_{i}\right\},I\right)=\frac{\sum_{P,Q}Z\left(P,Q\right)\;p\left(\theta |\left\{d_{i}\right\},P,Q,I\right)}{\sum_{P,Q}Z\left(P,Q\right)}\;\mathrm{so}\;\mathrm{that}\;\langle \theta^{M}\rangle \approx \frac{\sum_{P,Q,l}Z\left(P,Q\right)\;w_{P,Q}^{\left(l\right)}\;{\left[\theta_{P,Q}^{\left(l\right)}\right]}^{M}}{\sum_{P,Q,l}Z\left(P,Q\right)\;w_{P,Q}^{\left(l\right)}}.$$

Likewise, the posterior probabilities for the model orders considered jointly and separately are

(15) $$p\left(P,Q|\left\{d_{i}\right\},I\right)=\frac{Z(P,Q)}{\sum_{P,Q}Z\left(P,Q\right)};\;p\left(P|\left\{d_{i}\right\},I\right)=\frac{\sum_{Q}Z\left(P,Q\right)}{\sum_{P,Q}Z\left(P,Q\right)};\;p\left(Q|\left\{d_{i}\right\},I\right)=\frac{\sum_{P}Z\left(P,Q\right)}{\sum_{P,Q}Z\left(P,Q\right)}.$$

Finally, we note that sampling θ from Equation (13) instead of Equation (12) reduces the dimensionality of the parameter space from

nm+r+1=n(P+2Q)+2Q+1→r=2Q,

which compares favorably to the increased cost of evaluating $L_{I}$ compared to *L*. In a typical application, n=3, P=5 and Q=3, such that the dimensionality is reduced from 40 to a mere 6 dimensions. The dimensionality of the problem does not depend on the number of pitch periods *n* (but its complexity does).

### 3.1. The Integrated Likelihood

We begin by writing Equation (12) as

(16) $$\begin{array}{cc} Z(P,Q) & =\int d\theta \int d\sigma \;p(\theta |I)\;p(\sigma |I)\\ \; & \;\times \prod_{i=1}^{n}\int db_{i}\;{\left(2\pi \sigma^{2}\right)}^{-N_{i}/2}\;{\left(2\pi \delta^{2}\right)}^{-m/2}\exp\{-\frac{Q_{F}^{\left(i\right)}}{2}\}, \end{array}$$

where the quadratic form for the *i*th pitch period

$$Q_{F}^{\left(i\right)}={\left(d_{i}-G_{i}\;b_{i}\right)}^{T}\;\frac{I}{\sigma^{2}}\;\left(d_{i}-G_{i}\;b_{i}\right)+\frac{1}{\delta^{2}}{b_{i}}^{T}\;\frac{I}{\delta^{2}}\;b_{i}.$$

The expression for the integrated likelihood $L_{I}$, defined implicitly by Equation (13), can be found from Equation (16) by marginalizing over the amplitudes $\left\{b_{i}\right\}$ and the standard deviation σ.

We begin with the $\left\{b_{i}\right\}$. Defining (see, e.g., App. A in [38])

$$\begin{array}{c} g_{i}={G_{i}}^{T}G_{i}\\ \hat{b_{i}}=\mathrm{solution}\;\mathrm{of}\;\{\frac{\partial }{\partial b_{i}}Q_{F}^{\left(i\right)}=0\}={g_{i}}^{-1}\;{G_{i}}^{T}\;d_{i}\\ \hat{f_{i}}=f\left(t_{i},\hat{b_{i}},\theta \right)=G_{i}\;\hat{b_{i}} \end{array}$$

it can be shown after some effort that (using, e.g., [50])

(17) $$Q_{F}^{\left(i\right)}≃{\left(b_{i}-\hat{b_{i}}\right)}^{T}\;\frac{g_{i}}{\sigma^{2}}\;\left(b_{i}-\hat{b_{i}}\right)+\frac{{d_{i}}^{T}d_{i}}{\sigma^{2}}-\frac{{\hat{f_{i}}}^{T}\hat{f_{i}}}{\sigma^{2}}+\frac{{\hat{b_{i}}}^{T}\hat{b_{i}}}{\delta^{2}}.$$

This is a good approximation if $\left(g_{i}/\sigma^{2}+I/\delta^{2}\approx g_{i}/\sigma^{2}\right)$, i.e., if for all j=1⋯m it holds that

$${\left[\frac{g_{i}}{\sigma^{2}}\right]}_{jj}\gg \frac{1}{\delta^{2}}\Leftrightarrow \sum_{k=1}^{N_{i}}{\left[\frac{G_{i}}{\sigma^{2}}\right]}_{kj}^{2}\gg \frac{1}{\delta^{2}}\Leftrightarrow \frac{\mathrm{integrated}\;\mathrm{power}\;\mathrm{of}\;\mathrm{the}\;j\mathrm{th}\;\mathrm{basis}\;\mathrm{function}}{\mathrm{noise}\;\mathrm{power}}\gg \frac{1}{\delta^{2}}.$$

In our implementation, where δ=1, we found this to be an acceptable approximation for all states $\left(\left\{b_{i}\right\},\theta ,\sigma \right)$ with an appreciable likelihood *L*.

It is interesting to note that when the least-squares measure

$$\chi_{i}^{2}\left(b_{i},\theta \right)={e_{i}}^{T}e_{i}={\left(d_{i}-G_{i}\;b_{i}\right)}^{T}\left(d_{i}-G_{i}\;b_{i}\right)$$

is evaluated at the optimal amplitudes $\hat{b_{i}}$, it reduces to

$$\chi_{i}^{2}\left(\hat{b_{i}},\theta \right)\equiv {\hat{\chi }}_{i}^{2}\left(\theta \right)={d_{i}}^{T}d_{i}-{\hat{f_{i}}}^{T}\hat{f_{i}}.$$

Thus, Equation (17) can be written from left to right as the sum of (a) a density term, (b) a term quantifying the goodness-of-fit and (c) a regularization term:

(18) $$Q_{F}^{\left(i\right)}≃{\left(b_{i}-\hat{b_{i}}\right)}^{T}\;\frac{g_{i}}{\sigma^{2}}\;\left(b_{i}-\hat{b_{i}}\right)+\frac{{\hat{\chi }}_{i}^{2}}{\sigma^{2}}+\frac{{\hat{b_{i}}}^{T}\hat{b_{i}}}{\delta^{2}}.$$

Having completed the square in $Q_{F}^{\left(i\right)}$, the integral over the amplitudes $b_{i}$ is elementary, and we arrive at

(19) $$\begin{array}{cc} Z(P,Q) & ≃\int d\theta \;p\left(\theta |I\right)\;[\int d\sigma \;p\left(\sigma |I\right)\;{\left(2\pi \sigma^{2}\right)}^{\frac{nm-N}{2}}\;\exp\{-\frac{\sum_{i=1}^{n}{\hat{\chi }}_{i}^{2}}{2\sigma^{2}}\}]\\ \; & \;\times {\left(2\pi \delta^{2}\right)}^{-nm/2}\prod_{i=1}^{n}{\left|\det g_{i}\right|}^{-1/2}\exp\{-\frac{{\hat{b_{i}}}^{T}\hat{b_{i}}}{2\delta^{2}}\}. \end{array}$$

When it comes to the polynomial amplitudes, marginalizing over them can be seen as detrending (also called background removal [41]). Likewise, marginalization over the damped sinusoid amplitudes corresponds to removing their amplitudes and phases, i.e., we are only interested in the poles.

The next step is to marginalize over the standard deviation by performing the integral in the large square brackets in Equation (19), i.e.,

(20) $$\frac{1}{\log(\sigma^{\mathrm{hi}}/\sigma^{\mathrm{lo}})}\int_{\sigma^{\mathrm{lo}}}^{\sigma^{\mathrm{hi}}}\sigma \;\frac{1}{\sigma }\;{\left(2\pi \sigma^{2}\right)}^{\frac{nm-N}{2}}\;\exp\{-\frac{\sum_{i=1}^{n}{\hat{\chi }}_{i}^{2}}{2\sigma^{2}}\}.$$

We assume a reasonable amount of model functions *m* in relation to the number of data points *N* such that N>nm. For states (θ,σ) with appreciable likelihood practically all of the mass of Equation (20) is concentrated near the peak of its integrand at

(21) $$\hat{\sigma }=\sqrt{\frac{\sum_{i=1}^{n}{\hat{\chi }}_{i}^{2}}{N-nm+1}},$$

which we may assume to be within the bounds $\left[\sigma^{\mathrm{lo}},\sigma^{\mathrm{hi}}\right]$ if these are sufficiently wide and a reasonable fit to the data is possible (see App. A in [39] for more details). Assuming this is the case, Equation (20) can be safely converted into an elementary gamma integral by letting $\sigma^{\mathrm{lo}}\to 0$ and $\sigma^{\mathrm{hi}}\to \infty$ and the marginalization can be performed analytically.

Doing so we finally obtain the expression for the integrated likelihood $L_{I}$:

$$\begin{array}{cc} Z(P,Q) & =\int d\theta \;L_{I}\left(P,Q,\theta \right)\;p\left(\theta |I\right)\\ \; & \mathrm{with}\;L_{I}\left(P,Q,\theta \right)≃C\left(P,Q\right)\times {\left[\sum_{i=1}^{n}{\hat{\chi }}_{i}^{2}\right]}^{\frac{nm-N}{2}}\times \prod_{i=1}^{n}{\left|\det g_{i}\right|}^{-1/2}\exp\{-\frac{{\hat{b_{i}}}^{T}\hat{b_{i}}}{2\delta^{2}}\}, \end{array}$$

where

$$C\left(P,Q\right)=\frac{1}{2}\frac{1}{\log(\sigma^{\mathrm{hi}}/\sigma^{\mathrm{lo}})}\pi^{\frac{nm-N}{2}}\;\Gamma \left(\frac{N-nm}{2}\right)\;{\left(2\pi \delta^{2}\right)}^{-nm/2}$$

is a pure model order regularization term, being function only of *P* and *Q*. The factor ${\left[\log\left(\sigma^{\mathrm{hi}}/\sigma^{\mathrm{lo}}\right)\right]}^{-1}$ due to the normalization of the Jeffreys prior for σ is a constant independent of (P,Q,θ) and subsequently cancels out in model averaging and the weighting of posterior samples of θ.

### 3.2. Optimization Approaches

Though the nested sampling approach proposed here is different from the optimization approach we used in the conference paper, it is still possible to formulate a straightforward iterative optimization scheme for this problem. Indeed, a least-squares search in θ can still be used—the marginalization over the amplitudes would then be called “variable projection” [51], and the amplitude regularization in Equation (18) can still be incorporated by treating the $\left\{b_{i}\right\}$ as model predictions for nm additional datapoints measured to be zero with errorbar δ. To fix the scale for the actual *N* datapoints, these could be assigned fictional errorbars as well with magnitude $\hat{\sigma }$ as defined in Equation (21). Fast Fourier transformations, initially on the data and after on fit residuals, could be used for the initial guesses for the formant frequencies [39].

However, we have refrained from developing this approach mainly because of two reasons. First, due to the low dimensionality of the problem the nested sampling runs we ran to calculate Z(P,Q) for the next section were not unbearably slow (even in a Python/NumPy context) as most runs finished under two minutes. Second, nested sampling allowed us to calculate the evidence for a model order Z(P,Q) with confidence, while an optimization approach based on a Laplace approximation can easily give poor results. We did not, however, consider variational approaches.

## 4. Application to Data

In our experiments we used Praat [52] interfaced with the parselmouth Python library [53] with the default recommended settings to segment steady-state vowels into *n* pitch periods and get initial estimates for the formant bandwidths and frequencies to determine plausible ranges for their true values (Table 1). For the nested sampling we used the static sampler of the excellent dynesty Python library [54], again with default settings.

The range of the model order *P* was generally set to P=(1,2,…,10) while the allowed values of *Q* were more specific to the application (given that the signal bandwidth was limited to 4 kHz). In our experiments we found that high-degree polynomials tend to become too wiggly, thereby competing against the damped sinusoids for spectral power in awkwardly high frequency regions. It appeared that a good rule of thumb to prevent this behavior was to limit P≤10 (and thus set the maximum polynomial degree to 9). We also note that the case P=1 together with n=1 would correspond to the Pinson model of Section 1.2, if we would disregard the fact that we model the entire pitch period (from GCI to GCI) as opposed to only the portion between GCI and GOI.

### 4.1. Synthesized Steady-State /*ɤ*/

We apply the model first to a synthetic steady-state vowel /ɤ/ to verify the model’s prediction accuracy and to see whether the inferred polynomial correlates with $u^{\prime }\left(t\right)$, which we would expect based on the arguments of Section 2.4. The vowel was generated with different parameter settings [32] (p. 121) which emulate female and male speakers at different fundamental frequencies $F_{0}$ spanning the entire range of normal (non-pathological) speakers [4].

The vowel /ɤ/ was synthesized by first generating an artificial glottal source signal—the glottal flow derivative $u^{\prime }\left(t\right)$—at a sampling rate of 16 kHz, which was then filtered by an all-pole VT transfer function consisting of $Q_{\mathrm{true}}=3$ poles with realistic formant values (which we will refer to as “the true values” from now on) based on Reference [55] (p. 163), and then downsampled to 8 kHz. Now yielding to the usual notation of acoustic phonetics, the true bandwidths $B_{\mathrm{true}}$ (our αs) and frequencies $F_{\mathrm{true}}$ (our ωs) used for the VT transfer function are $B_{\mathrm{true}}=\left(54,22,19\right)\;\mathrm{Hz}$ and $F_{\mathrm{true}}=\left(430,1088,2142\right)\;\mathrm{Hz}.$

The glottal flow derivative $u^{\prime }\left(t\right)$ was generated using the LF model [56]. For the male speakers, the 11 values of $F_{0}=\left(80,90,\ldots ,180\right)$ Hz, increasing in steps of 10 Hz. Likewise, for female speakers, the 11 values of $F_{0}=\left(160,170,\ldots ,260\right)$ Hz. We applied tiny but realistic values of jitter (0.5%) and shimmer (2%) to the generated pitch periods, which greatly improved the perceived naturalness of the steady-state vowel’s sound. Finally, we selected the n=3 central pitch periods for analysis.

As mentioned before, the range of *P* was set to P=(1,2,…,10). The allowed range of *Q* was set to Q=(1,2,3). The ranges for the formant bandwidths and frequencies $\left[\theta_{j}^{\mathrm{lo}},\theta_{j}^{\mathrm{hi}}\right]$ are given in Table 1.

**Figure 2:**
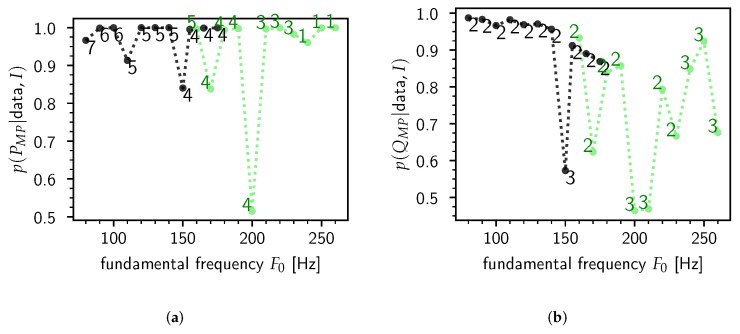
The most probable model orders $P_{\mathrm{MP}}$ and $Q_{\mathrm{MP}}$ and their posterior probability as calculated according to Equation (15). Each point in the graphs represents a synthesized steady-state /ɤ/ according to a speaker sex and fundamental frequency $F_{0}$. The sex is indicated by black (male) or lightgreen (female). The values of $P_{\mathrm{MP}}$ and $Q_{\mathrm{MP}}$ are indicated by text. **(a)** $p(P_{\mathrm{MP}}|ɤ,I)$. **(b)** $p(Q_{\mathrm{MP}}|ɤ,I)$.

For each synthesized steady-state /ɤ/, the posterior probability of *P* and *Q* was calculated—see Figure 2. It is clear that the majority of the most probable values $P_{\mathrm{MP}}$ and $Q_{\mathrm{MP}}$ are close to unity, which indicates that typically the model has an outspoken preference for a particular value of *P* and *Q*. In this case we see that $Q=2\neq Q_{\mathrm{true}}=3$ is heavily preferred, which means that in this particular experiment the model did not pick up the third formant [57]. Contrary to the number of formants *Q*, the preferred polynomial order P−1 is more dependent on variations in $F_{0}$, with *P* about 5 to 6 for male speakers and smaller for female speakers.

**Figure 3:**
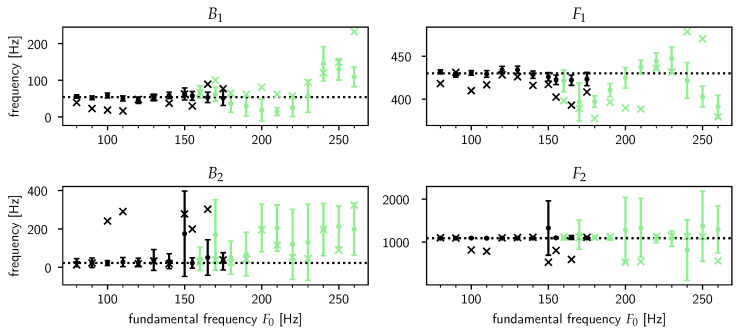
The model’s prediction accuracy for the bandwidth and frequency of the first $\left(B_{1},F_{1}\right)$ and second $\left(B_{2},F_{2}\right)$ formants. Each point in the graphs represents an estimate either by our model or by Praat for a synthesized steady-state /ɤ/ according to a speaker sex and fundamental frequency $F_{0}$. The sex is indicated by black (male) or lightgreen (female). The model’s estimates are averaged over all allowed model orders (i.e., values of (P,Q)) according to Equation (14), though in practice only one or two values of (P,Q) dominate (as suggested by Figure 2). The model’s estimates are the dots with the errorbars at three standard deviations. The linear predictive coding (LPC) estimates acquired with Praat are plotted as crosses. The true values $B_{\mathrm{true}}$ and $F_{\mathrm{true}}$ are drawn as dotted horizontal lines.

In Figure 3, the results of a test of the model’s prediction accuracy according to the model-averaged estimates in Equation (14) are shown for the frequency and bandwidth of the first $\left(B_{1},F_{1}\right)$ and second $\left(B_{2},F_{2}\right)$ formants. In accordance with Figure 2, we did not show the estimates for the third $\left(B_{3},F_{3}\right)$ formant as the most probable value of the number of formants in the data is $Q_{\mathrm{MP}}=2$—indeed, the errorbars for the estimates for $\left(B_{3},F_{3}\right)$ were huge, rendering those estimates practically useless.

Figure 3 shows a striking dichotomy between the results for male and female speakers. For the male speakers, the model’s estimates seem to perform equally well or better than the LPC estimates. In contrast, the performance dramatically decreases for female speakers with the true values often outside of the already exceedingly large error bars. For $B_{2}$ and $F_{2}$, the model does communicate its uncertainty about the true value of the bandwidths and frequencies by returning estimates with huge error bars, but unfortunately for $B_{1}$ and $F_{1}$ its estimates can be quite misleading.

The reason for this significant change in performance is mainly due to the change in the fundamental frequency $F_{0}$. As $F_{0}$ rises, the near-impulse response waveforms triggered by the GCIs tend to “spill over” into the next pitch period, i.e., the damped sinusoids caused by a given GCI are still ringing out appreciably when the next GCI happens, thus contaminating the pack of newly triggered decaying sinusoids. These nearest-neighbor effects increasingly wreck the assumption that a pitch period is only made up of a trend plus the VT impulse response. This is also evident from Figure 2a, where the most probable degree of the trend polynomial $\left(P_{\mathrm{MP}}-1\right)$ drops to zero for very high fundamental frequencies, suggesting that the low-frequency content is picked up by $F_{1}$, which Figure 3 confirms. From this experiment it appears that the threshold of $F_{0}$ is around 150 Hz. This essentially means that *the model is limited to male speakers only*.

The difficulties we encounter here reflect a known phenomenon in the literature: formant analysis for female voices is in general harder compared to male voices, regardless of the method used (e.g., [15], see also [16] (pp. 124–126) for an excellent discussion). Indeed, the negative correlation between $F_{0}$ and the estimates’ accuracy in Figure 3 is exhibited for both our model and Praat’s LPC algorithm. Next to the higher $F_{0}$ we already mentioned, another cause of this phenomenon is the fact that the coupling between the glottal source and VT is generally stronger in females [58], which violates the assumption of source and filter separability which underlies source-filter theory (“the female VT is not merely a small-scale version of the male VT” [59]).

**Figure 4:**
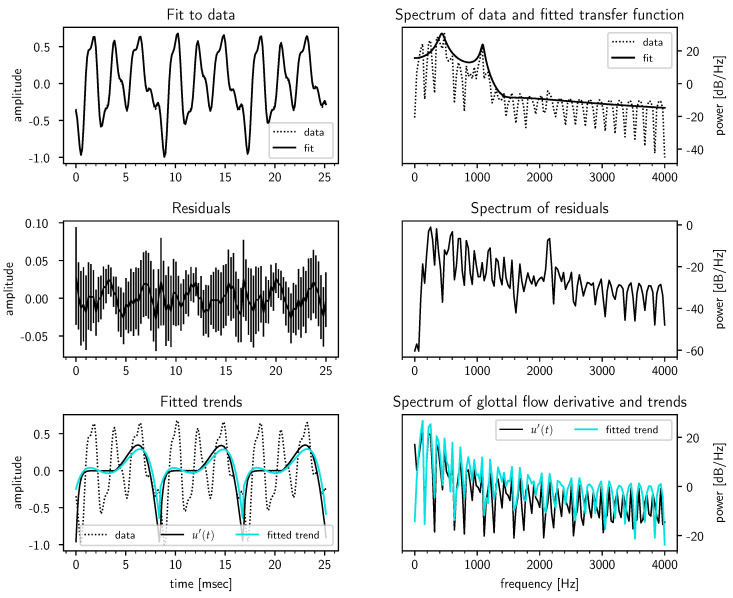
Fit results for a synthetic /ɤ/ in the case $F_{0}=120$ Hz for a male speaker. The fitted transfer function (solid line) in the top right panel is averaged over the n=3 pitch periods as the inferred vocal tract (VT) transfer functions can in general have different zeros and gain constants (but must share the same poles θ). The errorbars on the residuals in the center left panel are at three standard deviations.

Next, in Figure 4, we look at a typical case for male speakers, $F_{0}=120$ Hz [32], for which the model performed quite well. The third formant $F_{3}$ can be seen clearly in the spectrum of the residuals, though the model concluded it was noise as can be seen by the rather large error bars on the fit residuals. The bottom panels also show that the inferred polynomial trend correlates well with the true $u^{\prime }\left(t\right)$.

**Figure 5:**
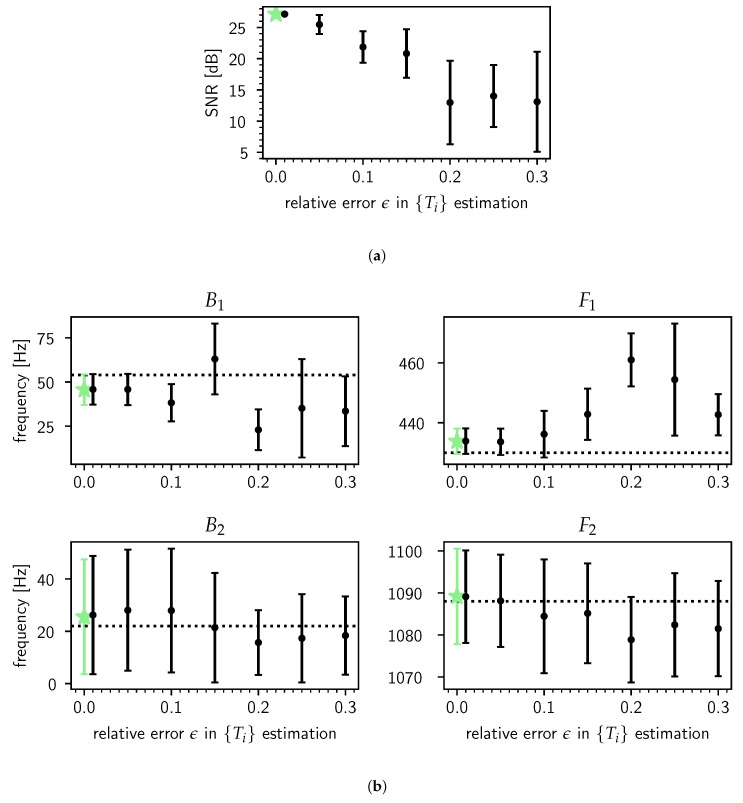
Testing the robustness of the bandwidth and frequency estimates of the first $\left(B_{1},F_{1}\right)$ and second $\left(B_{2},F_{2}\right)$ formants against increasing relative error ϵ in pitch period $\left\{T_{i}\right\}$ estimation for a synthetic /ɤ/ in the case $F_{0}=120$ Hz for a male speaker. Errors in $\left\{T_{i}\right\}$ estimation induce errors in the pitch period segmentation according to the GCIs $\left\{\tau_{i}\right\}$ and thus transfer to the formant estimates, which are acquired through model averaging as defined in Equation (14). The method is explained in detail in the main text. In each panel the green star indicates the estimates for the unperturbed $\left\{\tau_{i}\right\}$ (i.e., ϵ=0%), for which no averaging has been done. (**a**) The fit quality as gauged by the SNR (defined in Section 2) as a function of ϵ. Each point and its errorbar are the empirical mean and standard deviation at three σ, respectively, over 6 draws. For reference, the *prediction gain* of adaptive LPC for stationary voiced speech sounds is typically about 20 dB [60] (p. 70). (**b**) Comparison of the formant estimates as a function of ϵ to their true values $B_{\mathrm{true}}$ and $F_{\mathrm{true}}$ (dotted horizontal lines). Each point is the empirical mean of the point estimates over 6 draws, and each errorbar is the empirical mean of the point estimates’ *errorbars* at three standard deviations over the same 6 draws.

Finally, using the same synthesized steady-state vowel as in Figure 4, we gauge how inaccuracies in the segmentation of the steady-state vowel into *n* pitch periods affect the model’s estimates. In Figure 5, we simulate errors in this preprocessing step by parametrizing the relative error in estimating the pitch periods $\left\{T_{i}=\tau_{i+1}-\tau_{i}\right\}$ as ϵ (0≤ϵ≤1) and perturb the n+1 known GCIs at $\left\{\tau_{i}\right\}$ according to

$$\tau_{i}\to \tau_{i}^{\left(\epsilon \right)}=\tau_{i}+\left[T_{i}-T_{i}^{\left(\epsilon \right)}\right]\;\mathrm{where}\;\log T_{i}^{\left(\epsilon \right)}∼N\left(\log T_{i},\epsilon \right)\;\left(1\leq i\leq n+1\right).$$

The steady-state vowel is then segmented into *n* pitch periods $\left\{{d_{1}}^{\left(\epsilon \right)}\cdots {d_{n}}^{\left(\epsilon \right)}\right\}$ using the set of perturbed GCIs $\left\{\tau_{i}^{\left(\epsilon \right)}\right\}$ and estimates of the noise amplitude ${\hat{\sigma }}^{\left(\epsilon \right)}$ and the ${\hat{\theta }}^{\left(\epsilon \right)}$ are obtained. We repeated this procedure 6 times by drawing 6 sets of the $\left\{T_{i}^{\left(\epsilon \right)}\right\}$ for each value of ϵ∈{1%,5%,10%,15%,…,30%}. The results averaged over the draws are shown in Figure 5a,b. The conclusion from this particular experiment is that while the fit quality deteriorates strongly as the relative error in estimating the pitch periods grows (a), the formant estimates degrade relatively gracefully (b). One contributing factor for this is a feature of our model: it performs a kind of generalized averaging [39] (Sec. 7.5) over the pitch periods to arrive at “robust” estimates of the θ.

### 4.2. Real Steady-State /æ/

For real data, we do not know the underlying glottal source $u^{\prime }\left(t\right)$ as this is very hard to measure reliably. An alternative to measuring the glottal flow directly is the electroglottograph (EGG). The EGG signal can be used as a probe for the glottal source, as we will explain below.

The CMU ARCTIC database [29] consists of utterances which are recorded simultaneously with an EGG signal. The source of the steady-state /æ/ used in this section is a male speaker called BDL, file bdl/arctic_a0017.wav, 0.51–0.54 s, resampled to 8000 Hz. The fundamental frequency $F_{0}$ is about 138 Hz.

Once again the range of *P* was set to P=(1,2,…,10). The allowed range of *Q* was set to Q=(1,2,3,4). The ranges for the formant bandwidths and frequencies $\left[\theta_{j}^{\mathrm{lo}},\theta_{j}^{\mathrm{hi}}\right]$ are given in Table 1.

**Figure 6:**
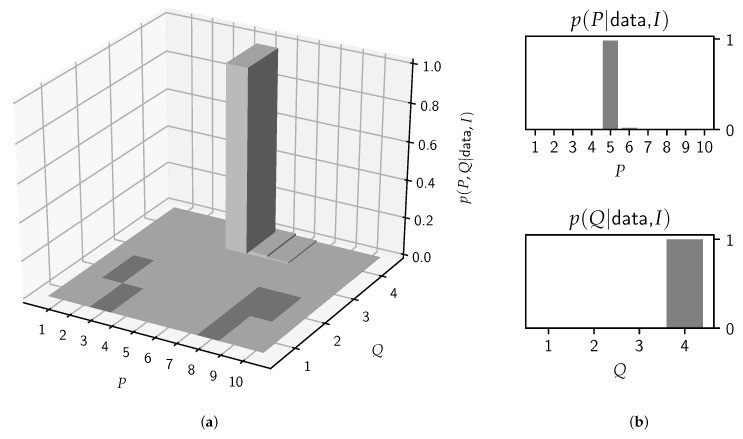
The posterior probabilities of the joint (**a**) and separate (**b**) model orders for the steady-state /æ/ according to Equation (15). In this case, model averaging is for all practical purposes equivalent to model selection as the model (P=5,Q=4) occupies 98% of the posterior mass.

In Figure 6, the posterior probability for the individual model orders is shown, from which a clear preference for P=5 and Q=4 arises.

**Figure 7:**
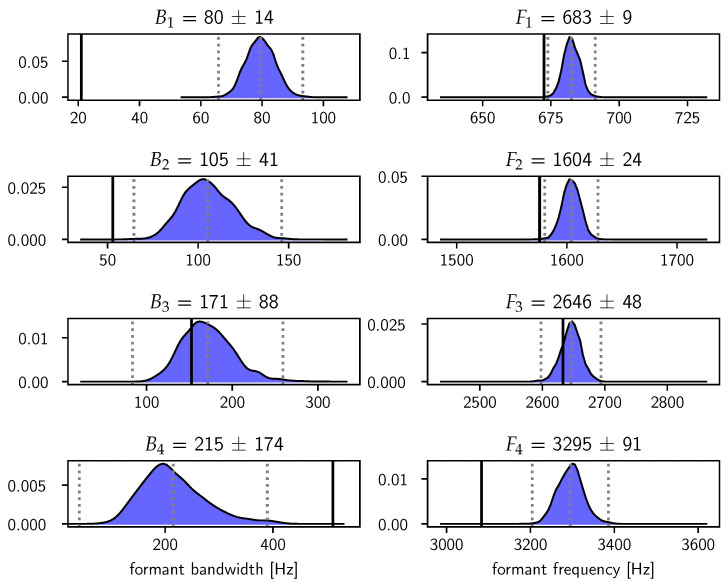
Posterior distributions p(θ|æ,I) of the formant bandwidths B and frequencies F. The distributions are estimated using Gaussian kernel density estimation for the combined samples $\theta_{P,Q}^{\left(l\right)}$ which are reweighted according to $w_{P,Q}^{\left(l\right)}\to w_{P,Q}^{\left(l\right)}\times Z\left(P,Q\right)/\sum_{P,Q}Z\left(P,Q\right)$. The dotted vertical lines indicate a distance of three standard deviations from the mean, which is also stated in the panel titles together with the point estimate. The solid vertical lines indicate the LPC estimates obtained with Praat.

In Figure 7, we show the model-averaged posterior distributions in Equation (14) for the formant bandwidths and frequencies. The estimates of the frequencies are reasonably sharp and agree quite well with the LPC estimates obtained with Praat. The error bars on the bandwidths increase gradually until the uncertainty on $B_{4}$ has become so large that it is essentially unresolved. This increase in the uncertainty on the bandwidths mirrors the fact that measuring bandwidths becomes increasingly difficult for higher formants [13].

**Figure 8:**
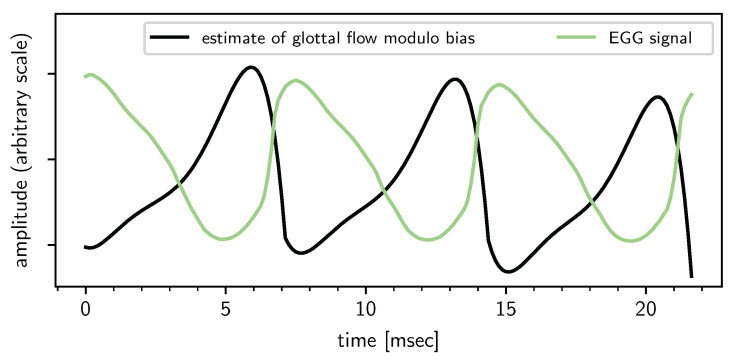
Comparison of the estimate of u(t) modulo a bias constant and the measured electroglottograph (EGG) signal. The speech signal, and therefore the u(t) estimate, lags behind the EGG signal by approximately 1 ms due to the distance between the glottis and the microphone.

Finally, we correlate the inferred trend together with the EGG signal in Figure 8. The EGG signal is the electrical conductance between two electrodes placed on the neck. When the glottis is closed and the glottal flow u(t) is zero, the measured conductance is high, and vice versa. The EGG signal rises sharply at the GCI, i.e., when the glottal flow drops abruptly to zero. From the discussion in Section 2.4 and in particular Equation (10), the glottal flow u(t) modulo a bias constant can be estimated very roughly from the inferred polynomial by integrating it over time. It is seen in the plot that the expected anticorrelation is borne out: when the u(t) estimate hits a through (GCI), the EGG signal rises sharply. Conversely, when the u(t) estimate hits a peak (GOI), the EGG signal hits a through, as the electrical conductance across the two electrodes drops due to the opening of the glotts.

## 5. Conclusions

The proposed model is a modest step towards formant estimation with reliable uncertainty quantification in the case of steady-state vowels. In our approach, the uncertainty quantification is implemented through Bayesian model averaging. The validity of our approach depends on the assumption that pitch periods can be modeled accurately as being composed of a slowly changing trend superimposed on a set of decaying sinusoids that represent the impulse response of the VT. It appears that this assumption likely holds only for fundamental frequencies $F_{0}$ below about 150 Hz, which poses a grave restriction on its use as this excludes most female speakers.

## Figures and Tables

**Table 1:** The prior ranges $\left[\theta_{j}^{\mathrm{lo}},\theta_{j}^{\mathrm{hi}}\right]$ for the Jeffreys priors for θ used in the two applications of the model to data. The ranges for the formant bandwidths ($\alpha_{j}$) and frequencies ($\omega_{j}$) are given in Hz; for example, the prior range for the first formant $\omega_{1}$ is 200–700 Hz. The data consists of a synthetic steady-state /ɤ/ (Section 4.1) and a real steady-state /æ/ (Section 4.2).

	$\alpha_{1}$		$\alpha_{2}$		$\alpha_{3}$		$\alpha_{4}$		$\omega_{1}$		$\omega_{2}$		$\omega_{3}$		$\omega_{4}$	
/ɤ/	10	180	10	250	10	420	/		200	700	700	1500	1500	3000	/	
/æ/	40	180	40	250	60	420	60	420	300	900	1000	2000	2000	3000	2500	4000
